# Decreased Functional Connectivity of Homotopic Brain Regions in Chronic Stroke Patients: A Resting State fMRI Study

**DOI:** 10.1371/journal.pone.0152875

**Published:** 2016-04-13

**Authors:** Chaozheng Tang, Zhiyong Zhao, Chuang Chen, Xiaohui Zheng, Fenfen Sun, Xiaoli Zhang, Jing Tian, Mingxia Fan, Yi Wu, Jie Jia

**Affiliations:** 1 Department of Rehabilitation Medicine, Huashan Hospital, Fudan University, Shanghai, China; 2 Shanghai Key Laboratory of Magnetic Resonance, East China Normal University, Shanghai, China; 3 College of Rehabilitation Medicine, Fujian University of Traditional Chinese Medicine, Fuzhou, China; 4 School of Psychology and Cognitive Science, East China Normal University, Shanghai, China; 5 Department of Rehabilitation Medicine, Jingan District Center Hospital, Shanghai, China; The University of Melbourne, AUSTRALIA

## Abstract

The recovery of motor functions is accompanied by brain reorganization, and identifying the inter-hemispheric interaction post stroke will conduce to more targeted treatments. However, the alterations of bi-hemispheric coordination pattern between homologous areas in the whole brain for chronic stroke patients were still unclear. The present study focuses on the functional connectivity (FC) of mirror regions of the whole brain to investigate the inter-hemispheric interaction using a new fMRI method named voxel-mirrored homotopic connectivity (VMHC). Thirty left subcortical chronic stroke patients with pure motor deficits and 37 well-matched healthy controls (HCs) underwent resting-state fMRI scans. We employed a VMHC analysis to determine the brain areas showed significant differences between groups in FC between homologous regions, and we explored the relationships between the mean VMHC of each survived area and clinical tests within patient group using Pearson correlation. In addition, the brain areas showed significant correlations between the mean VMHC and clinical tests were defined as the seed regions for whole brain FC analysis. Relative to HCs, patients group displayed lower VMHC in the precentral gyrus, postcentral gyrus, inferior frontal gyrus, middle temporal gyrus, calcarine gyrus, thalamus, cerebellum anterior lobe, and cerebellum posterior lobe (CPL). Moreover, the VMHC of CPL was positively correlated with the Fugl–Meyer Score of hand (FMA-H), while a negative correlation between illness duration and the VMHC of this region was also detected. Furthermore, we found that when compared with HCs, the right CPL exhibited reduced FC with the left precentral gyrus, inferior frontal gyrus, inferior parietal lobule, middle temporal gyrus, thalamus and hippocampus. Our results suggest that the functional coordination across hemispheres is impaired in chronic stroke patients, and increased VMHC of the CPL is significantly associated with higher FMA-H scores. These findings may be helpful in understanding the mechanism of hand deficit after stroke, and the CPL may serve as a target region for hand rehabilitation following stroke.

## Introduction

Stroke is the primary cause of prolonged disability in China and results in a heavy burden on patients and caregivers [1]. After stroke, 80% of survivors suffer from upper limb weakness and only approximately 30% of patients fully recovery [2]. The self-care level of hemiplegic patients mainly relies upon their upper limbs, especially the hands, which have a vital role in individual’s daily living and social participation [3]. Generally, spontaneous recovery following brain injury often is maximal within the first six months after insult [4, 5]. Recent studies have demonstrated that non-invasive brain stimulation, such as transcranial direct current stimulation (tDCS) and transcranial magnetic stimulation (TMS) [6, 7], were promising rehabilitation techniques for chronic stroke patients with hand dysfunction; however, the stimulation protocol for possible brain area targeting is deficient due to the limited knowledge concerning the underlying recovery mechanisms of brain reorganization in stroke patients [8–10].

Stroke often occurs in the dominant areas of the middle cerebral artery unilaterally, thereby generating the abnormal change of remote brain networks, which may contribute to both the disconnection and imbalance of the hemispheres [11]. Prior findings have suggested that the alterations of functional connectivity (FC) in the homotopic primary motor cortex (M1) during resting state and fractional anisotropy in the transcallosal M1–M1 tract were both correlated with the upper extremity function in the acute and chronic stage of stroke [12–14]. Moreover, extensive regions beyond the primary lesion showing degeneration of white matter integrity, including the ipsilesional medial frontal gyrus, superior temporal gyrus, supplementary motor area, and bilateral postcentral gyrus, have been reported in chronic stroke patients [15]. This may provide the structural basis of disrupted inter-hemispheric FC in the whole brain. However, so far, the resting-state FC between homotopic regions of the whole brain in chronic stroke patients is rarely reported. Considering the importance of bilateral hemispheric processing for behavioral performance, it is meaningful to examine inter-hemispheric coordination in stroke patients.

The low frequency oscillations (0.01–0.08 Hz) of blood-oxygen-level-dependent signals during rest were demonstrated to reflect spontaneous neuronal activities by functional magnetic resonance imaging (fMRI) [16]. And, this could provide a novel approach for the detection of intrinsic characteristics of geometrically symmetrical regions. Moreover, as a salient feature of the indwelling brain architecture [17], the homotopic resting-state FC (RSFC) has been shown to be available in the auditory, visual, and sensorimotor systems [18, 19] and may offer a sensitive index for the motor-related changes after stroke. Recently, a new and robust measure established by Zuo et al [20, 21] named voxel-mirrored homotopic connectivity (VMHC), which could quantify the RSFC between each voxel and its counterparts in two homologous cortices. While applying VMHC, abnormal RSFC for subjects with pathological states such as, schizophrenia [22], autism [23], depression [24], and cocaine addiction [25], have been reported in several recent studies. These studies suggested that VMHC might be regarded as a reliable measure for the detection of behavior-associated, bi-hemispheric alterations in stroke patients.

Here, we first explored the differences of VMHC between the chronic stroke patients and healthy controls (HCs) to evaluate the alterations of RSFC in bilateral mirrored regions. Based upon evidence from previous neurological diseases [11–14, 22–25], which have shown functional disconnection and asymmetry, we hypothesized that the inter-hemispheric FC might be impaired in patients with hand dysfunction following stroke. Moreover, we also explored whether, within the chronic stroke patients, there was a relationship between the VMHC of each survived regions and clinical tests.

## Materials and Methods

### Subjects

**Fig 1:**
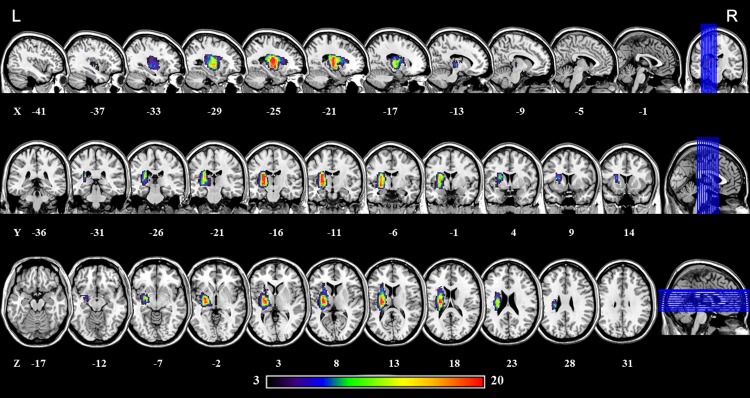
Lesion overlap map across the 29 stroke patients. Color bar indicates the number of subjects had lesion in each voxel.

A total of 67 participants, including 30 first-episode left subcortical stroke patients and 37 age-, gender-, and handedness-matched HCs were recruited from December 23, 2014 to August 7, 2015 through one ongoing prospective observational study (Study on Clinical Standardized Therapeutic Regimen and Evaluation System of TCM Rehabilitation in Hand Dysfunction after Stroke, ChiCTR-TRC-14004232). The inclusion criteria were as follows: (1) first onset subcortical stroke verified by computed tomography or magnetic resonance imaging (MRI); (2) age from 40 to 75 years; (3) apoplexy duration more than 3 months; (4) pure motor deficit and Brunnstrom classification of hand less than grade VI; and (5) right handedness before stroke, which was confirmed by modified Edinburgh Handedness Scale [26]. Exclusion criteria for the study included: (1) severe aphasia that impeded basic communication and examination; (2) any contraindications for MRI; (3) neurological disorders other than stroke, such as psychiatric disease; (4) unstable conditions, such as severe atrial fibrillation. Detailed demographics and clinical data of all patients are summarized in S1 Table, and lesion location of each patient, as determined by T2-weighted imaging, is shown in S1 Fig. We manually outlined the profiles on T2-weighted images slice by slice using the software MRIcron (www.mricro.com). And, maps of the lesion overlap for all stroke patients are shown in Fig 1. There were no significant differences between groups in age (t = 0.30, df = 64, p = 0.76), or gender (χ^2^ = 1.02, df = 1, p = 0.31) (See Table 1).

**Table 1:** Demographic and clinical data of all subjects.

	Stroke patients(n = 30)	Healthy controls(n = 37)	Statistic	P
	Mean±SD	Mean±SD	value	value
Gender(male/female)	25/5	27/10	χ^2^_1_ = 1.02	0.31
Age(years)	61.06±6.77	60.24±8.52	t_64_ = 0.30	0.76
Illness duration(months)	17.86±18.22	-		
Lesion location	L,SC(30), C(0)	-		
Stroke type	I(15), H(15)	-		
Lesion volume (ml)	40.37±34.98	-		
FMA-H score	5.41±4.63	-		

Abbreviations: L = left; SC = subcortical stroke; C = cortical stroke; I = infarction; H = hemorrhage; FMA-H = Fugl–Meyer Scale of hand section.

The Ethics Review Board of Huashan Hospital approved this work. The study procedures were conducted in accordance with the Declaration of Helsinki, and written informed consent was obtained from all participants.

### Image Acquisition

The imaging was implemented on a Siemens Trio 3.0 Tesla MRI scanner (Siemens, Erlangen, Germany) at Shanghai Key Laboratory of Magnetic Resonance. Resting-state fMRI scans were obtained using an echo-planar imaging sequence: 30 axial slices, 4 mm thickness, 0.8 mm gap, TR/TE 2000/30 ms, 90° flip angle, 220 mm^2^ FOV, 64×64 matrix, 240 volumes, and acquisition time lasted for 8 min 6 s (the first 6 s was dummy scanning). High-resolution structural images were acquired by employing a magnetization-prepared rapid gradient echo sequence:192 sagittal slices, 1 mm thickness, 0.5 mm gap, TR/TE/TI 1900/3.42/900 ms, 240 mm^2^ FOV, 9° flip angle, and 256×256 matrix. T2-weighted images were collected to identify the lesions using a turbo-spin-echo sequence: 30 axial slices, 5 mm thickness, no gap, TR/TE 6000/93 ms, 220 mm^2^ FOV, 120° flip angle, and 320×320 matrix. During scanning, all participants were instructed to remain awake, keep their eyes closed and rest without thinking about anything in particular.

### Data Preprocessing

Preprocessing of the imaging data were performed using Statistical Parametric Mapping (SPM8, http://www.fil.ion.ucl.ac.uk/spm) and Data Processing Assistant for Resting-State fMRI (DPARSF, http://resting-fmri.sourceforge.net). Primarily, we conducted the following steps: (1) the first 10 volumes were discarded to allow for magnetization equilibrium and environment adaptation; (2) the time delay between slices and rigid-body head movement during scans were corrected. Subjects with greater than 2.0 mm of translation or 2.0 degree of rotation in any direction were removed. One patient was excluded due to excessive head motion, and 29 patients and 37 controls were included for further analysis; (3) after motion correction, each T1 image was co-registered to the mean functional image, then were segmented into gray matter, white matter and cerebrospinal fluid; (4) using a unified segmentation algorithm [27] and the lesion mask, the functional images were normalized to the Montreal Neurological Institute space following motion correction and was then re-sampled to a 3×3×3 mm^3^ voxel using these parameters estimated during unified segmentation; (5) spatial smoothing of the normalized images were then performed using a 6 mm full width half maximum Gaussian kernel; (6) temporally bandpass filter (0.01–0.08 Hz) and linearly detrended removal were then conducted. Additionally, eight nuisance variances, including six head motion parameters, along with the signals from those regions centered in the ventricle and white matter were removed from data by linear regression.

### VMHC Calculation

VMHC was calculated by REST software (http://resting-fmri.sourceforge.net). The individual VMHC maps were converted to z values using a Fisher z-transformation to improve the normality. Furthermore, computational details of VMHC have been explicated by Zuo et al [20]. The individual z-maps were entered into a random effect two-sample t-test with the global VMHC as covariate in a voxel-wise manner to identify the difference in VMHC between groups (FDR corrected, P<0.05 and cluster>20).

### Correlation Analysis

Regions that demonstrate significant group differences between patients and HCs were used to evaluate relationships between the mean VMHC and the Fugl–Meyer score of hand (FMA-H), as well as the illness duration in stroke patients using a Pearson correlation analysis. All clinical tests of stroke patients were evaluated by a clinician from our department who was blinded to this study.

### Seed-based RSFC

To further study the alterations of functional networks behind the impaired VMHC, the brain areas that showed significant correlation between VMHC and clinical tests were selected as the regions of interest (ROIs). A voxel-wise FC analysis of each ROI was performed for the preprocessed fMRI data. For each subject and each seed region (namely ROI), a FC map of the whole brain was obtained by computing the correlation coefficients between the averaged time series of seed region and the time series of the remaining whole brain voxels. Correlation coefficients were converted to z values using Fisher’s r-to-z transformation to improve the normality. Then, a voxel-wise two-tailed two-sample t test was used to detect the difference of the RSFC maps between patients and HCs by using a whole gray matter mask (FDR corrected, p<0.05 and cluster>20).

## Results

### Difference between groups in VMHC

**Fig 2:**
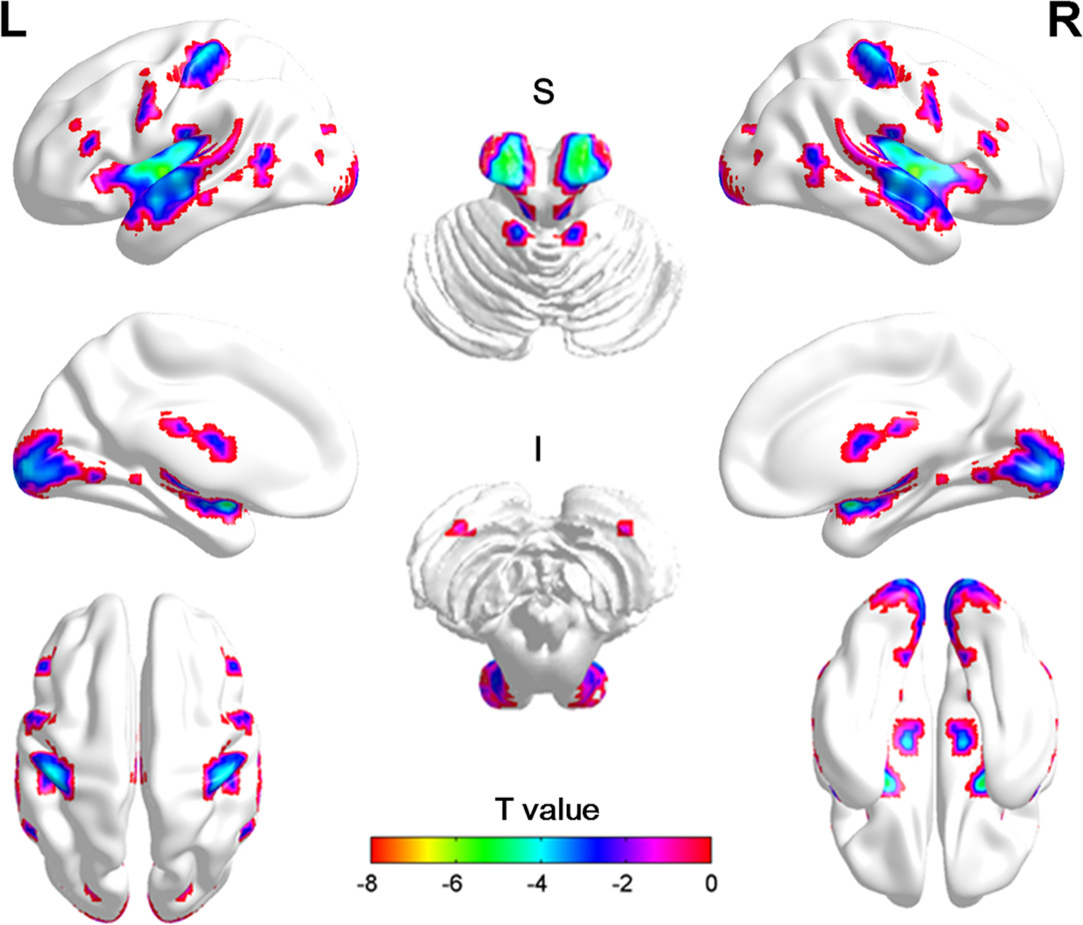
Regions showing significant differences in VMHC between groups. Cold colors indicate regions with a lower VMHC in chronic stroke patients when compared to healthy controls. The threshold was set at a FDR corrected p< 0.05, cluster>20. The color bar indicates the T value from the t-test between groups. VMHC: voxel-mirrored homotopic connectivity; S: superior; I: inferior.

Group comparisons revealed that chronic stroke patients showed significantly lower VMHC values than did HCs in the bilateral precentral gyrus, postcentral gyrus, inferior frontal gyrus (IFG), middle temporal gyrus (MTG), calcarine gyrus, thalamus, cerebellum anterior lobe (CAL), and cerebellum posterior lobe (CPL), as shown in Table 2 and Fig 2. Additionally, no regions showed greater VMHC in the patient group than in the control group. We excluded the thalamus from our study because this region partially overlaps with the lesion in stroke patients.

**Table 2:** Regions showing decreased VMHC in chronic stroke patients versus healthy controls.

Region	BA	MNI coordinate			Cluster	T value
		X	y	z		
**Patients < Controls**						
Precentral Gyrus	9	-51/51	6	39	102	-3.34
Postcentral Gyrus	3	-45/45	-24	57	271	-4.05
Inferior Frontal Gyrus	46	-48/48	30	15	43	-3.00
Middle Temporal Gyrus	39	-54/54	-57	9	33	-2.86
Calcarine Gyrus	17	-18/18	-96	-3	874	-4.25
Thalamus	-	-12/12	-15	9	4326	-7.67
Cerebellum Anterior Lobe	-	-9/9	-42	-9	32	-3.60
Cerebellum Posterior Lobe	-	-27/27	-78	-54	52	-3.17
**Patients > Controls**						
None						

Note: BA = Brodmann area; MNI = Montreal Neurological Institute; VMHC = voxel-mirrored homotopic connectivity.

### Correlations between VMHC and clinical variables

**Fig 3:**
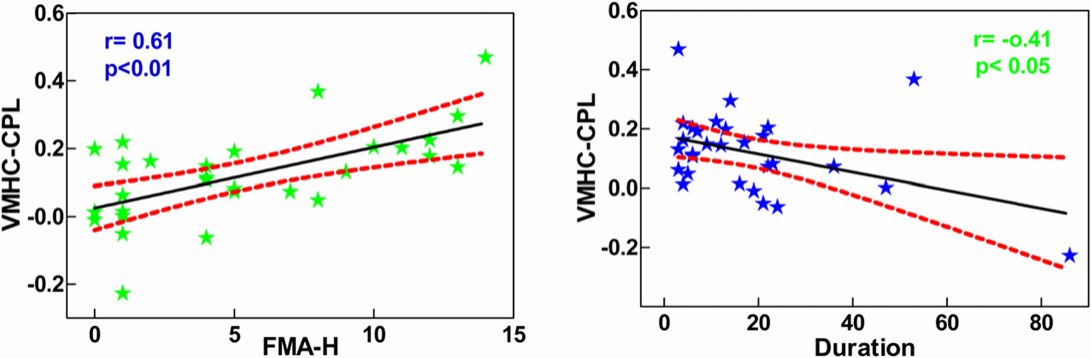
The significant correlations between the VMHC in the CPL and FMA-H scores as well as illness duration in patient group. VMHC: voxel-mirrored homotopic connectivity; CPL: cerebellum posterior lobe; FMA-H: Fugl–Meyer Score of hand.

The mean VMHC values were extracted from seven cortical areas that demonstrated significant group differences. Pearson correlations were performed between the VMHC and illness duration as well as FMA-H scores within the patient group. As shown in Fig 3, a significant positive relationship was observed between the VMHC in the CPL and the FMA-H scores (r = 0.61, p<0.01) in the patient group. And, a significant negative correlation between the illness duration and the VMHC in the CPL was also observed (r = -0.41, p<0.05).

### Seed-based FC

**Fig 4:**
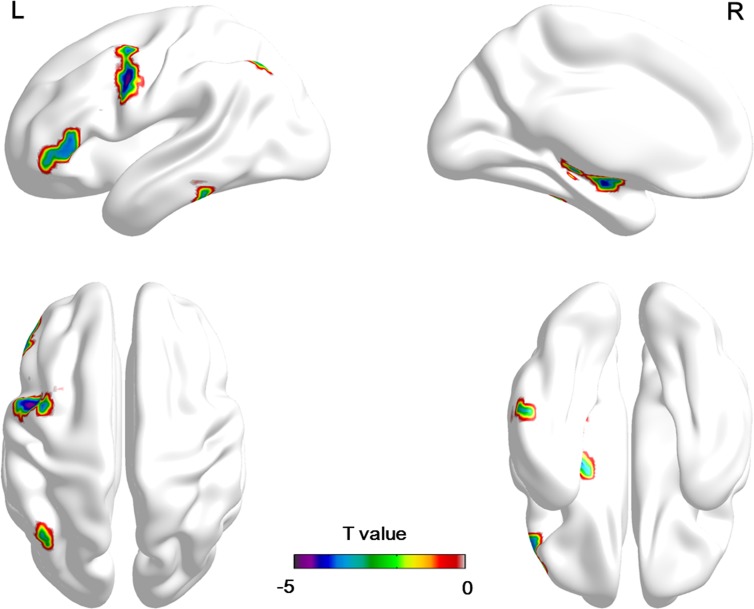
Significant differences in seed-based functional connectivity between chronic stroke patients and healthy controls. Cold colors indicate that these regions show a lower FC with the right CPL in chronic stroke patients when compared to healthy controls. The threshold for seed-based FC was set at a FDR corrected p< 0.05, cluster>20. The color bars indicate the T value from the t-test between groups. FC: functional connectivity; CPL: cerebellum posterior lobe.

As shown above, VMHC of the bilateral CPL exhibited significant correlation with the FMA-H scores and illness duration. We further selected the left CPL and right CPL as seed region to perform the whole-brain RSFC analysis. Relative to the HC group in chronic stroke patients, the right CPL exhibited reduced FC with the left precentral gyrus, IFG, inferior parietal lobule (IPL), MTG, thalamus and hippocampus, whereas no regions showed significant difference of FC with the left CPL (See Table 3 and Fig 4).

**Table 3:** Regions showing significant differences of functional connectivity between the CPL and the voxels of the whole brain.

Region	BA	MNI coordinate			Cluster	T value
		X	y	z		
**Right CPL**						
Patients < Controls						
Left Precentral Gyrus	6	-51	6	42	183	-4.73
Left Inferior Frontal Gyrus	47	-51	33	6	55	-3.89
Left Inferior Parietal Lobule	40	-42	-63	48	20	-3.86
Left Middle Temporal Gyrus	20	-69	-36	-18	51	-4.79
Left Thalamus	-	-18	-21	3	22	-4.21
Left Hippocampus	-	-27	-9	-9	130	-4.89
**Left CPL**						
None						

Note: BA = Brodmann area; MNI = Montreal Neurological Institute; CPL = cerebellum posterior lobe.

### Half verification

To confirm the reliability of our study, we randomly selected 15 patients and 18 controls and repeated the current results. Subsequent analyses showed that the difference of VMHC in the most brain regions between groups was still significant (uncorrected, p< 0.001 and cluster > 20), but the CPL not survived. All details are shown in S2 Table and S2 Fig.

## Discussion

Although the RSFC between the homotopic motor-related regions has already been investigated in other stroke studies [11–14], they did not take into account the VMHC changes of the whole brain networks. In the present study, we first applied VMHC to study abnormal connectivity of homologous areas in whole brain for chronic stroke patients. The main findings of this work are: (1) relative to HCs, the VMHC of the patient group was significantly reduced in the precentral gyrus, postcentral gyrus, IFG, MTG, calcarine gyrus, thalamus, CAL, and CPL; (2) a positive correlation was detected between the VMHC and FMA-H scores, and a negative relationship was observed between the VMHC and illness duration in the CPL; (3) the right CPL exhibited decreased FC with the left precentral gyrus, IFG, IPL, MTG, thalamus and hippocampus.

A recent fMRI review suggested that the assessment of homotopic FC between regions of motor-network which was performed using resting-state fMRI seem to be the clinically most promising way for the prognosis of motor recovery after stroke [14]. Carter and colleagues [12] reported that the disruption of homologous RSFC in the somatomotor network was significantly correlated with upper limb impairment. In contrast, intra-hemispheric FC within the normal or damaged hemispheres was not correlated with performance in either network. Similarly, Chen et al [13] also found that RSFC between the homotopic M1 after stroke correlated with upper extremity scores of FMA. In addition, a longitudinal fMRI study showed that the dynamic change in the inter-hemispheric RSFC between the bilateral primary sensorimotor cortexes (SMC) in stroke patients was positively correlated with the Motricity index [28]. Consistent with previous findings that showed motor deficit following stroke related to the disturbed homotopic FC in the sensorimotor network during rest [11–14, 28], we also found a reduced VMHC in the precentral gyrus and postcentral gyrus. Our current work extended these findings by showing that bi-hemispheric coordination in the Primary SMC was interfered in stroke patients, thereby producing inadequate inter-hemispheric communication and a disturbance in sensory or motor processing, which manifests as a motor deficit.

The prefrontal cortex (PFC), including the middle frontal gyrus and inferior frontal gyrus, which plays a central role in the planning and integration of the cognitive load that is required to execute motor performance [29]. A task-evoked fMRI study has demonstrated that the early activation of PFC and parietal cortex in the contralesional hemisphere predicted a slower and worse motor recovery [30]. What is more, in the stroke patients with poor outcome recovery, extra recruitment of the non-primary motor regions during simple tasks, like the PFC, was the bad indicator of motor recovery [8, 31]. Using the resting-state fMRI, our previous work have manifested that regional homogeneity (ReHo) in the ipsilesional PFC was increased significantly in severe chronic stroke patients when compared with mild ones. Additionally, a significantly negative correlation between the ReHo index in the ipsilesional medial frontal gyrus and FMA scores has been detected [32]. Moreover, a recently longitudinal study has shown that RSFC of the ipsilesional M1 with the contralesional middle frontal gyrus at onset was positively correlated with motor recovery at six months following stroke [33]. In the present study, we observed decreased VMHC between the homotopic IFG. Our work provides a new perspective for illustrating the role of IFG in motor impairment after chronic stroke, which implies that the abnormal RSFC of the bilateral IFG may impede the execution of motor tasks. Additionally, when compared with HCs, we observed that stroke patients showed decreased inter-hemispheric connectivity in the MTG and calcarine gyrus. These findings indicate that the change of homotopic FC underlying the motor recovery after a focal motor pathway stroke may even extend to the brain areas beyond the traditional motor-related networks [34].

It has been widely accepted that the cerebellum is involved in neural processes other than motor control, and the activation of contralesional anterior cerebellum 20 days after stroke has been found positively correlated with the motor performance of finger-tapping [35]. Based upon the available evidence concerning the cerebellum, the CAL (lobules I-V) and CPL (lobule VIII) predominantly contain the sensorimotor cerebellum, whereas lobules VI and VII of the CPL constitute the cognitive cerebellum [36–38]. In addition, the existence of cortico-ponto-cerebellar circuit indicates that information from prefrontal and associative sensorimotor cortices is processed in the CPL [37]. In the present study, we detected a lower inter-hemispheric RSFC of the CAL and CPL in chronic stroke patients, and a significant positive correlation was found between the VMHC of CPL and FMA-H scores. Therefore, we speculated that the VMHC of the CPL was a robust predictor of motor performance for stroke patients during chronic stage. It is possible that the CPL has a central role in the pathophysiology of chronic stroke and could serve as a rehabilitative target region for improving the hand function recovery in chronic stroke patients. Recently, using the resting-state fMRI, Hu et al found a reverse relationship between the VMHC of bilateral supramarginal gyrus and illness duration in Parkinson disease [39]. Similarly, we also found that the VMHC in the CPL negatively correlated with disease duration, indicating that the inter-hemispheric interaction of this region in stably chronic stroke patients was still influenced by illness course due to the learned non-use.

It was clear that a peak in the ipsilateral CAL (lobule V) and a second cluster in CPL (lobule VIII) were activated during sensorimotor tasks, whereas cognitive tasks were engaged in the prefrontal and parietal cortices along with lobules VI and VII of the CPL [37, 40, 41]. Moreover, several RSFC studies have suggested that the activity in sensorimotor areas were correlated with activity in CAL (lobules V) and CPL (lobules VIII), while cerebellar lobule VI of the CPL participates in functional networks with the PFC [42, 43]. In line with these findings [42, 43], we also found a significantly decreased FC of the right CPL with the left precentral gyrus, IFG, IPL, MTG, thalamus, and hippocampus in the patient group as compared to the HCs. These findings suggest that the abnormal FC of the right CPL, with associative sensorimotor and prefrontal cortices, may contribute to alterations in functional networks that result in impaired VMHC between the bilateral CPL.

While our results are interesting and encouraging, some limitations should be noted in the current study. First, the half verification is failed to examine the difference of VMHC in the CPL between groups and it may due to the small sample size. Therefore, a larger sample should be used to confirm this result. Second, the bias caused by the asymmetry of the brain structure should be considered. We recruited only left subcortical stroke patients to minimize the variable of inter-hemispheric architecture and applied an asymmetric standard template [44] to raise the methodological symmetry. Third, the exact substrate behind the reduced VMHC after a focal lesion has not been clear. Future studies may solve this by combining objective detections, such as DTI and TMS. Finally, due to the lack of longitudinal observation, the pattern by which these areas with a lower VMHC change dynamically during the clinical course of stroke requires further exploration.

## Conclusions

In summary, the current work shows that the RSFC between widespread homologous brain areas in patients with chronic stroke was impaired. More importantly, in the patient group, increased VMHC of the CPL was observed to be significantly associated with higher FMA-H scores and shorter illness duration. Our results are the first to reveal that the VMHC of the CPL could serve as a useful predictor for hand dysfunction after chronic stroke. These findings enable us to target therapies using non-invasive brain stimulation (tDCS/TMS) in motor rehabilitation poststroke that may modulate the inter-hemispheric coordination of bilateral CPL and thus enhance hand recovery.

## Supporting Information

S1 Fig Lesion location of the 30 stroke patients enrolled in this study. (TIF)

S2 Fig Statistical maps indicate VMHC difference between chronic stroke patients and healthy controls, which could be repeated by half verification. (TIF)

S1 Table Clinical and demographic data of the 30 stroke patients enrolled in this study. (DOCX)

S2 Table Regions showing significant differences in VMHC between chronic stroke patients and healthy controls could be clarified by half verification. (DOCX)
