# Supplementary figures and images for: Decreased Functional Connectivity of Homotopic Brain Regions in Chronic Stroke Patients: A Resting State fMRI Study
Source: PLoS One. 2016 Apr 13;11(4):e0152875. doi: 10.1371/journal.pone.0152875 (PMC4830618; doi:10.1371/journal.pone.0152875)

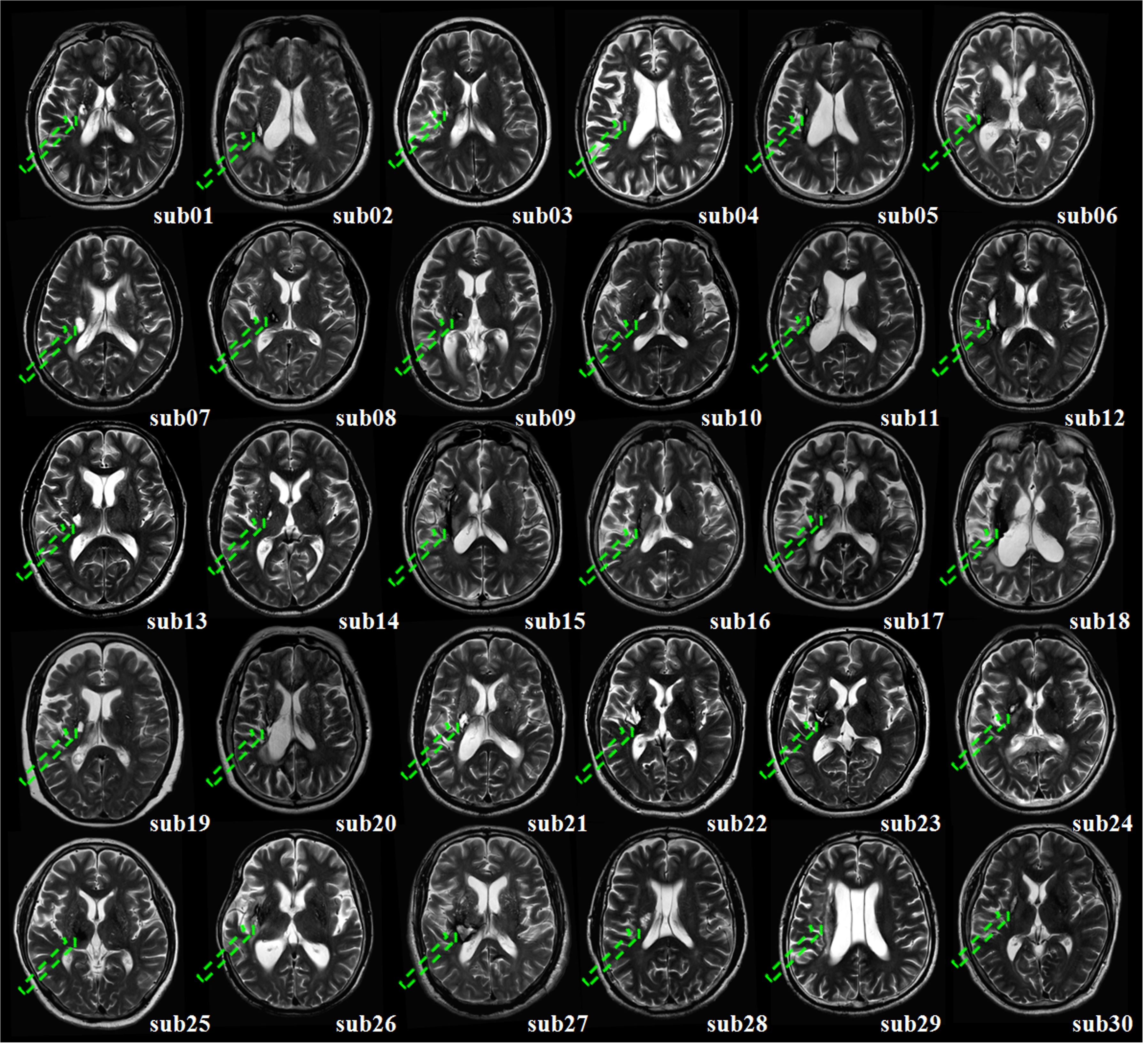

Supplement: S1 Fig — (TIF) [file pone.0152875.s001.tif]

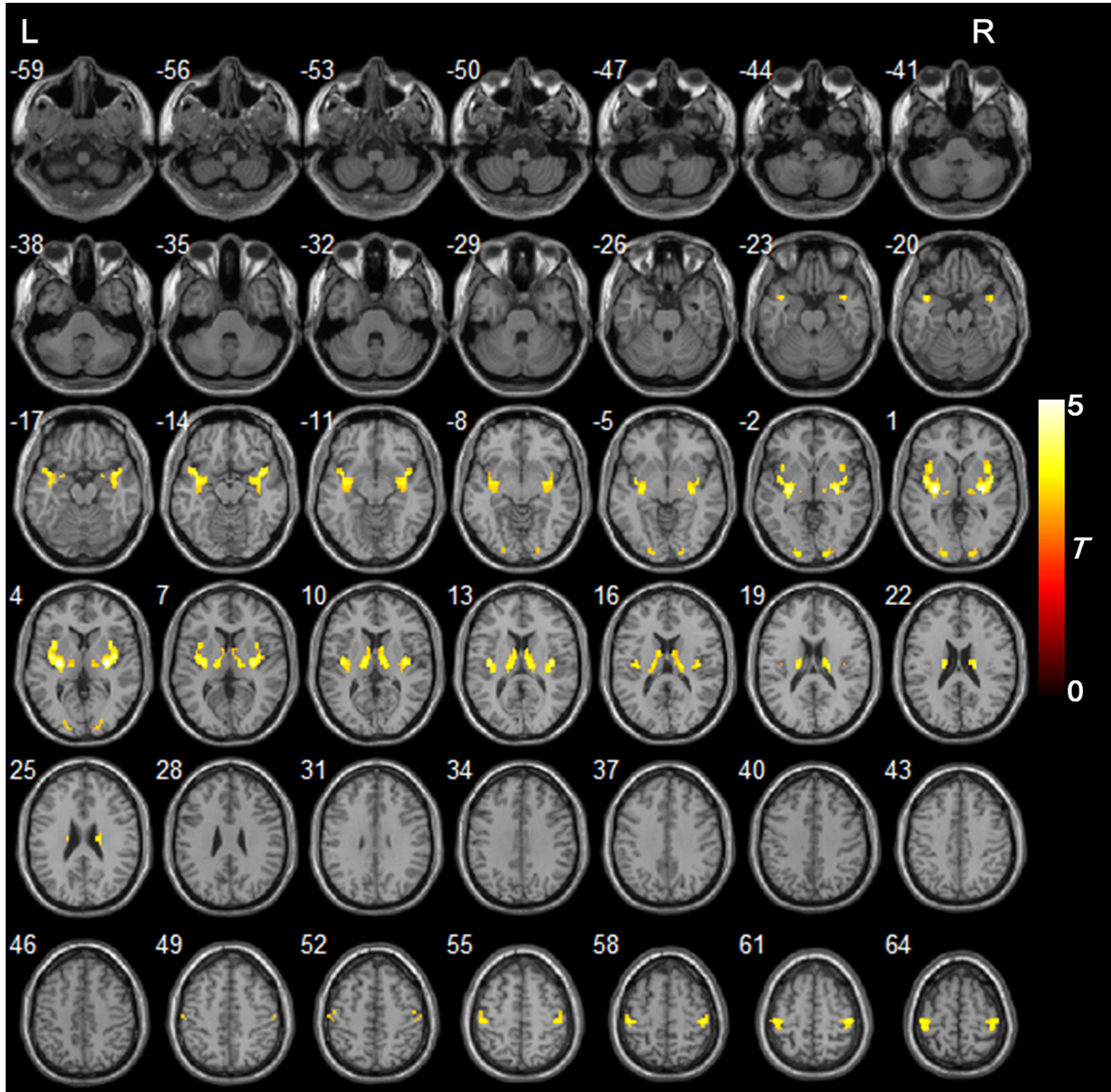

Supplement: S2 Fig — (TIF) [file pone.0152875.s002.tif]
