# Supplementary material for: Decreased Functional Connectivity of Homotopic Brain Regions in Chronic Stroke Patients: A Resting State fMRI Study
Source: PLoS One. 2016 Apr 13;11(4):e0152875. doi: 10.1371/journal.pone.0152875 (PMC4830618; doi:10.1371/journal.pone.0152875)
Supplement: S1 Table — (DOCX) [file pone.0152875.s003.docx]

**S1 Table. Clinical and demographic data of the 30 stroke patients enrolled in this study.**

| **Case** | **Gender** | **Age**  **(years)** | **Location of lesion** | **Time poststroke (months)** | **Lesion**  **volume(ml)** | **FMA score**  **(hand)** |
| --- | --- | --- | --- | --- | --- | --- |
| 01 | M | 56 | L,IC,BG,Th | 14 | 25.1 | 13 |
| 02 | M | 60 | L,IC,Th^a^ | 53 | 35.3 | 8 |
| 03 | F | 48 | L,IC,BG | 23 | 9.4 | 5 |
| 04 | M | 76 | L,IC | 21 | 62.2 | 12 |
| 05 | M | 60 | L,IC,BG | 36 | 16.5 | 5 |
| 06 | M | 71 | L,IC,Th^a^ | 22 | 17.6 | 7 |
| 07 | M | 63 | L,IC,BG,Th | 3 | 22.1 | 9 |
| 08 | M | 54 | L,IC,Th^a^ | 3 | 12.4 | 14 |
| 09 | M | 60 | L,BG,Th^a^ | 11 | 27.0 | 12 |
| 10 | M | 65 | L,IC,Th | 12 | 8.9 | 13 |
| 11 | M | 53 | L,IC,BG,Th^a^ | 22 | 125.1 | 10 |
| 12 | M | 65 | L,IC,BG^a^ | 6 | 81.8 | 11 |
| 13 | M | 62 | L,BG,IC,Th | 6 | 19.2 | 4 |
| 14 | M | 56 | L,IC | 7 | 10.6 | 5 |
| 15 | M | 56 | L,BG,IC,Th^a^ | 21 | 86.9 | 1 |
| 16 | M | 57 | L,IC,Th | 19 | 23.7 | 0 |
| 17 | F | 75 | L,IC,CR | 24 | 7.8 | 4 |
| 18 | M | 63 | L,BG,IC,Th^a^ | 16 | 61.5 | 1 |
| 19 | F | 65 | L,IC,Th | 17 | 29.6 | 1 |
| 20@ | F | 68 | L,BG,IC,Th^a^ | 62 | 25.5 | 0 |
| 21 | M | 68 | L,IC,Th^a^ | 47 | 32.7 | 1 |
| 22 | M | 53 | L,BG,IC,Th^a^ | 86 | 55.5 | 1 |
| 23 | F | 50 | L,BG,IC,Th^a^ | 13 | 18.7 | 0 |
| 24 | M | 61 | L,IC,BG | 6 | 9.7 | 4 |
| 25 | M | 56 | L,IC,Th^a^ | 5 | 27.4 | 8 |
| 26 | M | 69 | L,IC,BG^a^ | 4 | 145.3 | 0 |
| 27 | M | 61 | L,IC,Th^a^ | 4 | 60.5 | 1 |
| 28 | M | 59 | L,IC,BG | 4 | 21.7 | 2 |
| 29 | M | 61 | L,IC,BG | 3 | 76.2 | 1 |
| 30 | M | 61 | L,IC,BG | 9 | 40.4 | 4 |

Note: M=male; F=female. L=left; R=right; BG=basal ganglia; IC=internal capsule; Th=thalamus; CR=coronal radiata; FMA=Fugl-Meyer Assessment. ^a^The character of the lesion is hemorrhage, others are ischemia; @case 20 was excluded for excessive head motion.
