# Supplementary material for: Decreased Functional Connectivity of Homotopic Brain Regions in Chronic Stroke Patients: A Resting State fMRI Study
Source: PLoS One. 2016 Apr 13;11(4):e0152875. doi: 10.1371/journal.pone.0152875 (PMC4830618; doi:10.1371/journal.pone.0152875)
Supplement: S2 Table — (DOCX) [file pone.0152875.s004.docx]

**S2 Table. Regions showing significant differences in VMHC between chronic stroke patients and healthy controls could be clarified by half verification.**

| **Region** | **BA** | **MNI coordinate** | | | **Cluster size** | **T** |
| --- | --- | --- | --- | --- | --- | --- |
|  | | X | y | z |  | |
| **Patients < Controls** |  |  |  |  |  |  |
| Postcentral Gyrus | 3 | -42/42 | -33 | 66 | 97 | -3.32 |
| Precental Gyrus | 4 | - | - | - | 25 | - |
| Superior Temporal Gyrus | 38 | -30/30 | -15 | 0 | 343 | -4.41 |
| Calcarine Gyrus | 17 | -18/18 | -96 | -3 | 36 | -2.88 |
| Thalamus | - | -21/21 | -18 | 21 | 136 | -3.73 |
| **Patients > Controls** |  |  |  |  |  |  |
| None |  |  |  |  |  |  |

Note: BA=Brodmann area; MNI=Montreal Neurological Institute; VMHC= voxel-mirrored homotopic connectivity.
